# Correlations between growth and wool quality traits of genetically divergent Australian lambs in response to canola or flaxseed oil supplementation

**DOI:** 10.1371/journal.pone.0208229

**Published:** 2019-01-03

**Authors:** Aduli E. O. Malau-Aduli, Don V. Nguyen, Hung V. Le, Quang V. Nguyen, John R. Otto, Bunmi S. Malau-Aduli, Peter D. Nichols

**Affiliations:** 1 Animal Genetics and Nutrition, Veterinary Sciences Discipline, College of Public Health, Medical and Veterinary Sciences, Division of Tropical Health and Medicine, James Cook University, Townsville, Queensland, Australia; 2 National Institute of Animal Science, Hanoi, Vietnam; 3 College of Economics and Techniques, Thai Nguyen University, Thai Nguyen, Vietnam; 4 College of Medicine and Dentistry, Division of Tropical Health and Medicine, James Cook University, Townsville, Queensland, Australia; 5 CSIRO Oceans & Atmosphere, Hobart, Tasmania, Australia; Tokat Gaziosmanpasa University, TURKEY

## Abstract

The correlations between growth and wool traits in response to canola and flaxseed oil supplementation were evaluated in Australian prime lambs. Sixty dual-purpose prime lambs including purebred Merino and crossbred lambs were allocated to one of five treatments of lucerne hay basal diet supplemented with isocaloric and isonitrogenous wheat-based pellets. Treatments were: no oil inclusion (Control); 2.5% canola oil; 5% canola oil; 2.5% flaxseed oil and 5% flaxseed oil, with lamb groups balanced by breed and gender. Each lamb was daily supplemented with 1kg of pellets and had free access to lucerne hay and water throughout the 7-week feeding trial, after a 3-week adaptation. Individual animal basal and supplementary pellet feed intakes were recorded daily, while body conformation traits, body condition scores and liveweights were measured on days 0, 21, 35 and 49. The lambs were dye-banded on the mid-side and shorn before commencing the feeding trial and mid-side wool samples were collected from the same dye-banded area of each lamb at the end of the experiment. Correlations between wool quality traits and lamb performance were non-significant (P>0.05). Oil supplementation had no detrimental effect on lamb growth and wool quality traits (P > 0.05). Gender significantly affected wither height gain and fibre diameter. There were significant interactions between oil supplementation and lamb breed on chest girth. The correlations between clean fleece yield (CFY) and other wool quality traits were moderate ranging from 0.29 to 0.55. Moderate to high correlations between fibre diameter (FD) and other wool quality traits were detected (0.46–0.99) with the strongest relationship between FD and wool spinning fineness (SF). The relationship between CFY and wool comfort factor (CF) were positive, while negative relationships between CFY and the others were observed. A combination of 5% oil supplementation and genetics is an effective and strategic management tool for enhancing feed efficiency and growth performance without negative effects on wool quality in dual-purpose lamb production. This is a good outcome for dual-purpose sheep farmers. It essentially means the absorbed nutrients in supplemented lambs yielded good growth performance without any detrimental impact on wool quality; a win-win case of nutrient partitioning into the synthesis of muscle and wool without compromising either traits.

## Introduction

The increased incidences of central nervous system disorders, cardiovascular diseases and cancers have been associated with high consumption of red meat [1, 2] with high levels of saturated (SFA) and low omega-3 polyunsaturated (n-3 PUFA) fatty acid contents [3]. Previous studies had demonstrated that n-3 PUFA content in meat products can be manipulated by supplementing ruminants with feeds enriched with n-3 PUFA dietary sources [4,5] that include fish, algae, oilseeds and their oils [6, 7]. The practical inclusion of marine products in ruminant supplements is unsustainable due to prohibitively high cost and scarcity [8, 9] as well as concerns regarding detrimental effects on sensory eating quality [10, 11]. Thus, oilseeds and their oils are considered as alternative and sustainable sources of n-3 PUFA. Canola and flaxseed oils contain an abundance of α- linoleic acid (ALA, 18:3n-3) [12] and have been of recent interest in a few feeding trials aiming to increase n-3 PUFA levels in lamb [13–15]. However, these investigations mainly focused on variations in meat fatty acid profiles of lambs fed vegetable oils. Research on growth and wool quality responses of lambs to dietary oils rich in ALA have received little attention. Furthermore, limited on-farm research has been conducted on the optimal supplementary levels, duration of feeding and correlations between wool and growth traits of lambs supplemented with these oils.

Due to decreased wool prices in the past two decades, the Australian sheep industry has adopted dual-purpose sheep systems with both wool and meat production goals [16]. Genetic management of animals for enhancing both growth performance and wool quality in dual-purpose sheep systems through crossbreeding strategies provides a cumulative and long-term alternative approach to nutritional manipulation[17]. The effects of sheep breed and gender on growth responses and wool traits have been reported [18, 19]. However, these studies were conducted under grazing conditions with seasonal variability in pasture supply. Studies simultaneously comparing growth and wool attributes between sheep breeds in intensive lamb production systems during finishing periods remain scarce. Whereas previous studies had investigated lamb growth rates and slaughter weights in response to energy-rich supplements in research stations without investigating associations with wool quality traits, the current study is unique in its integrated approach in a typical ‘real world’ on-farm, dual-purpose and intensive crossbred lamb finishing system. Its uniqueness is further justified by an attempt to fill a currently existing knowledge gap on the appropriate supplementary levels and associated impacts of flaxseed and canola oils on feed intake, growth and wool quality of Australian prime lambs from different genetic backgrounds. Growth performance and carcass characteristics as well as variations in the fatty acid profiles of edible tissues, plasma metabolites and sensory characteristics of meat from the same 60 lambs used in the present study were reported by Nguyen et al. [20, 21, 22].The study reported herein, investigates the correlations between growth and wool quality traits of Australian dual-purpose lambs in response to supplementation with graded levels of either canola oil or flaxseed oil based pellets in an on-farm intensive finishing management system. It also aimed to estimate residual phenotypic correlations within and between wool quality traits and lamb performance under the same management system.

## Materials and methods

Growth performance and carcass characteristics as well as variations in the fatty acid profiles of edible tissues, plasma metabolites and sensory characteristics of meat from the same 60 lambs used in the present study have been reported [20–22]. As described in detail previously [22], this on-farm research was conducted under the auspices of the Tasmanian Institute of Agriculture, Tasmania, Australia from June to August, 2014 utilising Claire Blackwood’s flock in Cressy, Northern Tasmania. The experimental design and protocols were approved by the University of Tasmania Animal Ethics Committee (Permit No. A13839) and followed 2013 Australian Code of Practice for the Care and Use of Animals for Scientific Purposes.

### Animals and experimental design

Single-born prime lambs weighing between 4.5 and 5.5 kg were subjected to on-farm best practice operations of marking, vaccination, castration and tail-docking at about 12–14 weeks of age when they were weaned. Sixty weaner ewe (n = 30) and wether (n = 30) lambs, 6–7 months old with an average liveweight (LWT) of 33.4 ± 0.7 kg and body condition score (BCS) of 2.7 ± 0.3 were utilised in this study. The completely randomised experimental design comprised 20 purebred Merinos (MxM), 20 Corriedale × Merino (CxM) and 20 White Suffolk × Corriedale (WxC) first-cross lambs with equal number of ewe and wether lambs represented in each breed. Each lamb was supplemented daily with 1 kg of isocaloric and isonitrogenous wheat-based pellets and randomly allocated to one of five treatments of 12 lambs per group, balanced by breed and gender. The treatments were: no oil inclusion (0O); 2.5% canola oil (2.5CO); 5% canola oil (5CO); 2.5% flaxseed oil (2.5FO) and 5% flaxseed oil (5FO) on dry matter basis. The supplemental pellet ingredients and chemical composition of the dietary treatments have been published [22]. Briefly, wheat was the major carrier ingredient in the pellets (465–551 g/kg) and the diets were formulated to have similar DM, CP and EE contents among the treatment pellets (P>0.05). The ME contents ranging from 10.8 MJ/kg DM to 11.1 MJ/kg DM were also similar among the five oil based supplementary pellets. The CP and ME contents of the basal diet of lucerne hay were 17.4% DM and 9.8 MJ/kg DM respectively.

Lambs were fed for 7 weeks after a three-week adaptation period and had unlimited access to lucerne hay and clean water. They were offered fresh feed at 09.00 hours after residual feed left-over had been weighed and removed.

### Feed intake and growth measurements

The amount of offered pellet and lucerne hay and residual left-over feeds were separately weighed daily to calculate feed intake. Representative feed samples were collected on days 0, 25 and 49 of the experimental period and stored at -20°C for subsequent analyses.

Lambs were weighed and their body conformations measured on days 0, 21, 35 and 49 of the experiment before receiving their daily ration. Liveweights (LWT) were measured using a calibrated Ruddweigh 3000XT Walk-Over weighing electronic scale. Lamb LWT on initial and final days were obtained to calculate average daily gain (ADG).

The body conformation measurements of chest girth (CG), body length (BL) and wither height (WH) were taken using a measuring tape as outlined in detail [19]. Body condition scores (BCS) were also subjectively determined on a scale ranging from 0 (emaciated) to 5 (obese) by feeling the layer of tissue (fat and muscle) at the short rib region using the thumb and fingers on the ribs [22]. All measurements were made while lambs were in a relaxed state and restrained, with heads comfortably erect and standing stably upon all four legs by the same researcher throughout the trial to minimize stress and variations.

### Wool sampling and analysis

All lambs were shorn a month before the commencement of the trial. At the end of the experiment, wool was clipped from a mid-side patch (10 cm by 10 cm) of each lamb by an experienced shearer using Oster-Sunbeam electric shears (Boca Raton, FL, USA), as described by Langlands and Wheeler [23]. Wool quality traits were commercially evaluated at the Australian Wool Testing Authority (AWTA) using Sirolan Laserscan (AWTA Limited, Melbourne, VIC, Australia). Wool quality traits included clean fleece yield (CFY), mean fibre diameter (FD), fibre standard deviation (FSD), comfort factor (CF), fibre curvature (FC) and spinning fineness (SF). Wool quality traits have been described in detail [24]. In brief, CFY, expressed as a percentage, refers to the fibrous content of wool. FD refers to the average width of a single cross section of wool fibre and it is widely acknowledged as the most important wool property when assessing wool quality and value [24]. FSD is a measurement of fibre diameter variation within a normal distribution [24]. CF is defined as the percentage of wool fibres with diameter below 30 μm [25]. FC describes crimp frequency as the number of crimps per unit of length. Low FC is associated with softness of handle or low resistance to compression of both raw and scoured wool [26]. SF is a measure of the performance of the fibre when spun into yarn and it takes FD of the wool sample into account. SF permits accurate comparison and estimation of wool processing speed, cost, and yarn evenness [27], and low SF wool is typically more desirable and financially rewarded. It is therefore a sensible alternative to FD, as a selection attribute for wool sheep [28].

### Feed chemical analysis

At the end of the experiment, all feed samples were defrosted, pooled by treatments, and ground through a 1-mm screen. Samples were dried in triplicates in a fan-forced oven to a constant weight at 65°C to determine dry matter (DM) content. Total Nitrogen (N) value was quantified using an elemental analyser (PE2400 Series II; Perkin-Elmer Corp, USA) and crude protein (CP) content was estimated by multiplying N by 6.25. Ether extract (EE) was determined using an ANKOM fat/oil extractor (ANKOM^XT15^; ANKOM Technology, USA). An ANKOM fibre analyser (ANKOM220; ANKOM Technology, USA) was used to measure acid detergent fibre (ADF) and neutral detergent fibre (NDF) contents. The samples were combusted in a furnace at 550°C for 5 hours to quantify ash content. Non-fibrous carbohydrates (NFC) was calculated as NFC = 100 − (CP + NDF + EE + Ash) [29]. A near infrared reflectance spectroscopy method was used to estimate metabolisable energy [30]. Details of the ingredients and chemical compositions of the experimental feeds have been published [22].

### Statistical analysis

Feed efficiency (FE) was computed as gram LWT gain per kilogram of DM of feed consumed [31]. Liveweight, body conformation measurements and BCS were transformed into changes (Δ) between the initial and final values for each of the traits over the duration of the feeding trial [22].

All collected data were analysed using the Statistical Analysis System software version 9.2 [32]. General linear model (PROC GLM) analyses were used to fit supplementation, sheep breed, gender and their second-order interactions as fixed effects and feed intake, growth performance and wool quality characteristics as dependent variables. The final statistical model used for the analysis was: Y = μ + O_*i*_ + B_*j*_ + G_*k*_ + (OB)_*ij*_ + (OG)_*ik*_ + (BG)_*ik*_ + e_*ijk*_ where Y = dependent variable, μ = overall mean, O_*i*_ = oil supplementation, B_*j*_ = breed, G_*k*_ = gender, brackets represent second-order interactions and e_*ijk*_ = residual error.

The means were obtained using the LSMEANS option. Significant differences and mean separations were performed using Tukey’s probability pairwise comparison tests. Significant effects were declared at P < 0.05. Pearson’s correlation coefficients between wool quality traits were also estimated using PROC CORR procedure in SAS and significance established using Bonferroni probability pairwise comparison test.

## Results

**Fig 1:**
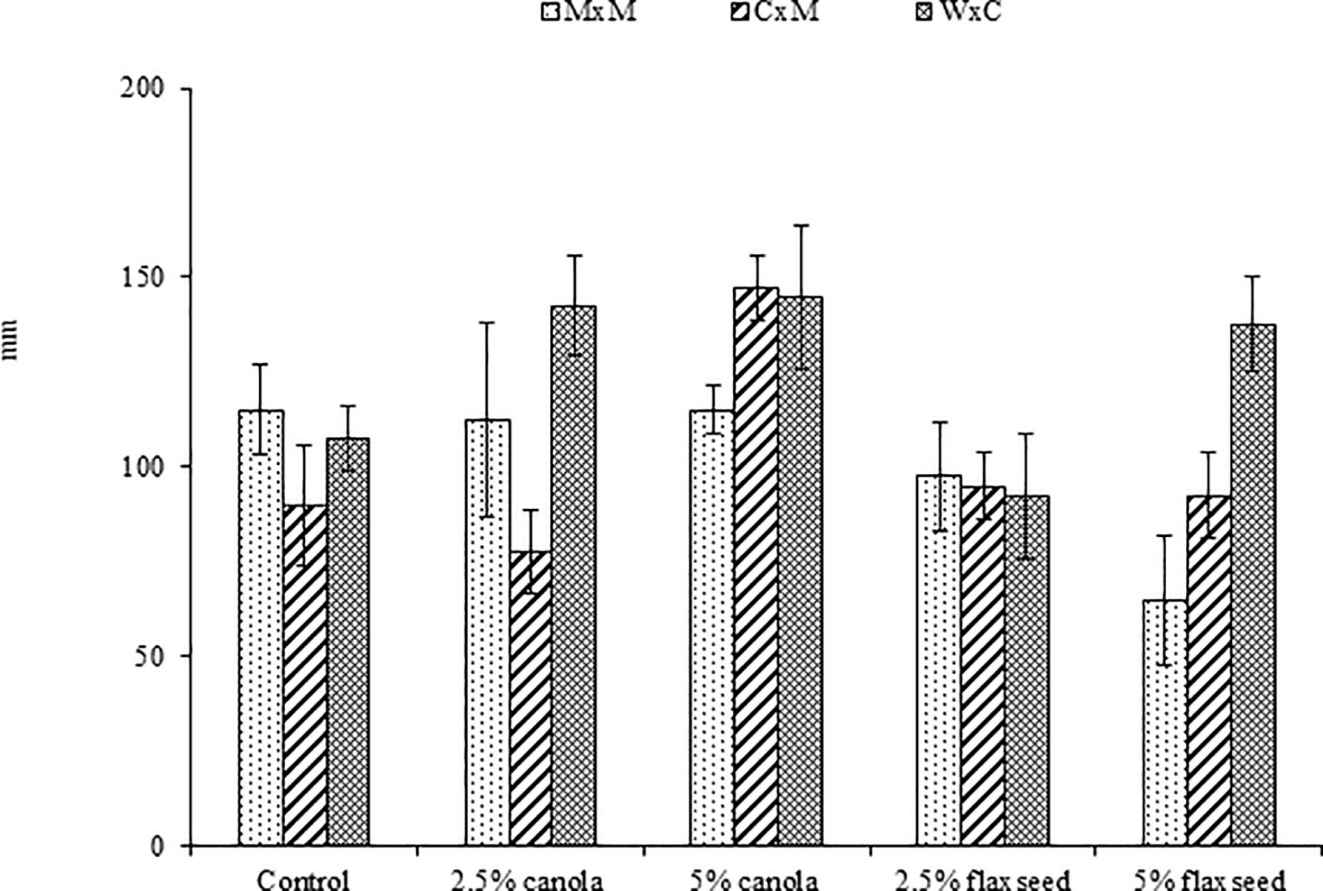
Interactions between omega-3 oil supplementation and breed on chest girth of experimental lambs (MxM: Merino x Merino; CxM: Corriedale x Merino; WxC: White Suffolk x Corriedale).

To be able to fully grasp the changes in liveweight, body conformation and condition score in this study, it is pertinent to refer readers to our previous publication where results of the dry matter intake, average daily gain, feed efficiency and body conformation measurements in this experimental flock were described in detail [22]. These details will not be repeated herein, except for Fig 1 depicting significant interactions between omega-3 oil supplementation and breed on chest girth of experimental lambs. The main focus in this current study is on new data on correlations between growth and wool traits.

### Wool quality attributes

Lamb breed was a significant source of variation in wool quality (P < 0.01; Table 1). Purebred Merino lambs had greater CFY (76.5%), CF (99.5%), and lower FD (17.4 μm), FC (53.5°/mm) and SF (16.4 μm) compared with first-cross lambs studied. Furthermore, variations in fibre diameter (FSD) were lesser in purebred Merinos than in crossbred lambs. Between the two crossbreds, similarity in wool quality traits were observed with the exception of FC. CxM lambs had lower FC (70.1°/mm) than WxC lambs (76.3°/mm) (P < 0.05). No significant second-order interactions on wool quality traits were detected. Fibre standard deviation was influenced by gender with ewes producing wool with greater FSD than wethers (P < 0.05; Table 1). However, gender had no significant effects on other wool quality traits. Wool quality traits were unaffected by the inclusion of canola and flaxseed oils in the pellets, compared with the control group (P > 0.05).

**Table 1:** Variation in wool quality as influenced by omega-3 oil supplementation, breed, gender and their interactions.

Item^1^	CFY (%)	FD (𝛍m)	FSD (𝛍m)	CF (%)	FC (°/mm)	SF (𝛍m)
**Treatment**						
**Control**	73.1	22.9	5.0	83.1	62.8	22.5
**2.5% canola**	72.7	23.0	4.5	84.7	67.2	22.1
**5% canola**	72.4	22.4	4.3	89.3	66.2	21.5
**2.5% flaxseed**	75.3	21.8	4.2	92.1	66.5	20.9
**5% flaxseed**	72.8	22.1	4.4	89.3	70.4	21.3
**Breed**^**2**^						
**MxM**	76.5^a^	17.4^b^	3.0^b^	99.5^a^	53.5^c^	16.4^b^
**CxM**	70.8^b^	25.3^a^	5.2^a^	79.7^b^	70.1^b^	24.6^a^
**WxC**	72.5^b^	24.7^a^	5.2^a^	83.9^b^	76.3^a^	24.0^a^
**Gender**						
**Ewes**	72.9	23.0	4.7^a^	85.9	68.3	22.3
**Wethers**	73.6	21.9	4.2^b^	89.6	64.9	21
**SEM**^**3**^	0.5	0.6	0.2	2.0	1.7	0.6
**P-values**						
**Treatment**	0.20	0.84	0.26	0.47	0.07	0.72
**Breeds**	0.01	0.01	0.01	0.01	0.01	0.01
**Sex**	0.44	0.15	0.05	0.30	0.14	0.10
Treatment*Breeds	0.58	0.77	0.91	0.81	0.51	0.85
Treatment*Sex	0.62	0.57	0.72	0.65	0.80	0.58
Sex*Breeds	0.85	0.55	0.15	0.68	0.16	0.40

^1^ CFY: clean fleece yield; FD: mean fibre diameter; FSD: fibre standard deviation; CV: coefficient of variation; CF: comfort factor; FC: fibre curvature; SF: spinning fineness.

^2^ MxM: purebred Merino; CxM: Corriedale x Merino; WxC: White Suffolk x Corriedale.

^3^ SEM: standard error of the mean.

^a,b,c^ means with different superscripts within a fixed factor significantly differ (P < 0.05).

### Correlations between wool quality traits and lamb performance

Table 2 illustrates that there were significant correlations between wool quality traits. The relationships between CFY and other wool quality traits were moderate ranging from 0.29 to 0.55. Moderate relationships between FC and the other traits were also observed. Moderate to high correlations between FD and other wool quality traits were detected (0.46–0.99) with the strongest relationship between FD and SF. The relationship between CFY and CF were positive, while negative relationships between CFY and the others were observed. As portrayed in Table 3, all the correlations between wool quality traits and lamb performance were non-significant (P>0.05).

**Table 2:** Pearson’s residual correlation coefficients between wool quality traits.

Item^1^	FD	FSD	CF	FC	SF
**CFY**	-0.43***^2^	-0.41**	0.32*	-0.55***	-0.44***
**FD**		0.91***	-0.91***	0.64***	0.99***
**FSD**			-0.84***	0.60***	0.94***
**CF**				-0.44***	-0.91***
**FC**					0.64***

^1^ CFY: clean fleece yield; FD: mean fibre diameter; FSD: fibre standard deviation; CV: coefficient of variation; CF: comfort factor; FC: fibre curvature; SF: spinning fineness.

^2^ * P < 0.05

** P < 0.01

*** P < 0.001.

**Table 3:** Pearson’s residual correlations between wool traits and lamb performance.

* *Item^1^	CFY	FD	FSD	CF	FC	SF
**ADG**	-0.29	0.23	0.17	-0.16	0.27	0.22
**LWG**	-0.29	0.23	0.17	-0.16	0.27	0.22
**CG**	0.06	0.13	0.14	-0.11	0.04	0.13
**BL**	-0.03	-0.13	-0.22	0.16	0.00	-0.15
**WH**	-0.01	0.13	0.16	-0.06	0.28	0.13
**BCS**	0.04	0.13	0.14	-0.15	0.10	0.14

^1^ADG: Average daily gain; LW: Liveweight gain; CG: Chest girth gain; BL: Body length gain; WH: Withers height gain; BCS: change in body condition score; CFY: clean fleece yield; FD: mean fibre diameter; FSD: fibre standard deviation; CF: comfort factor; FC: fibre curvature; SF: spinning fineness

## Discussion

The results of our previous findings from this same flock [22] were consistent with other studies [17, 22, 33–39] on growth traits related to meat production in lambs and had been presented in detail, hence will not be repeated herein. Apart from growth and meat production, wool products also contribute a significant percentage to economic returns in the dual-purpose sheep enterprise [40]. The additional data in the present study build upon previously published findings by providing new knowledge on the relationships between growth and wool parameters in the light of dietary oil supplementation, breed and gender effects. Herein, we illustrate that wool is not a uniform biological product because its physical characteristics vary depending on sheep genetics, environment and management strategies [41]. Wool fibres are primarily composed of protein [42], thus wool synthesis is frequently influenced by the quality and amount of dietary protein, especially sulphur-containing amino acids–cysteine and methionine [40]. However, protein content was not impacted by the inclusion of canola oil or flaxseed oil [22]. This could partly explain the fact that wool quality remained unchanged during the 10 weeks of oil supplementation. Additionally, the absence of significant differences in wool quality could be due to similarity in protein sources, forage to concentrate ratio and DMI among the dietary treatments [22]. This observation is in agreement with our previous study [17], in which wool quality was not impacted when canola oil was used as a supplement for finishing lambs. This is a good outcome for dual-purpose sheep farmers. It essentially means the absorbed nutrients in supplemented lambs yielded good growth performance without any detrimental impact on wool quality; a win-win case of nutrient partitioning into the synthesis of muscle and wool without compromising either traits.

Regarding gender effects, the difference between ewes and wethers in the present study was consistent with the findings of previous work [19, 43] and may be attributed to variation in body sizes between genders [44]. It has been demonstrated that ewes have a smaller mature size compared with male lambs because of the high oestrogen level restricting the growth of long bones including limb bones [45]. Ewes would be expected to have less CFY and FD than intact rams because they have less surface area, although the amount of follicles are similar between genders in the same breed which result in a greater follicle density [46]. However, in our study, gender did not affect the wool traits except for FSD which may be due to hormonal effects which impact metabolic pathways [18]. Wethers have lesser testosterone levels than intact rams and this can influence wool traits [16].

As previously demonstrated [22], the differences in CG among breeds in this study were in agreement with those reported in other studies [19, 43]. Furthermore, the wool quality differences between purebred Merinos and crossbred lambs in this current study were consistent with [18] who found purebred Merino lambs had lower FD, FC, SF and greater CF compared with crossbred-Merino lambs. Purebred Merinos had also been reported to have lesser FD and greater CFY than crossbred-Merino lambs [47]. A possible explanation for these differences includes variation in genetic disposition towards muscle growth, wool growth or body fat deposition [48, 49] and the diversity in production type among breeds [50]. The three breeds chosen in this study represented a variety of common genotypes in the Tasmanian and Australian sheep industry. Purebred Merino lambs represent a breed typically selected for wool production but also frequently used as a maternal breed in crossbred prime lamb production, whereas WxC and CxM crossbred lambs represent commercial dual-purpose prime lamb production. Lambs from high growth breeds grew larger than their counterparts from low growth pedigrees [44]. Another possible explanation for differences in wool quality traits is variation in wool follicle density between breeds [51]. Therefore, various studies have supported the observation that lamb breed is a significant source of variation in growth performance [18–20, 49].

It was interesting that significant interactions between nutrition and breed on lamb growth traits detected in Fig 1 and in our previous study [22] were widely acknowledged in published literature [19, 52, 53]. These provide lamb producers with a wider range of choices of nutritional regimen and breed combinations for targeting the reduction of feed cost and the optimal attainments of slaughter weights [19]. The nutritional and genetic interactions for lamb growth traits may allow the development of lamb production strategies to suit a spectrum of market specifications [52].

The correlations between wool quality traits in the present study are in accordance with preceding published literature [18, 54]. The very strongly positive relationship between FD and SF is logical and expected because SF is refined from FD and CV [29]. CF is used to describe the percentage of fibres with FD less than 30 **𝛍**m [19]. It means that increasing CF is attributable to a decrease in FD. Hence, it was concluded that there were highly negative correlations between CF and both FD and SF [25]. It has also been reported that the two measurements of variation in FD (FSD and CV) had strongly positive correlations, although there was a moderate correlation between FD and FSD [54]. These findings are in line with the results in this study.

## Conclusions

Supplementing pellets containing up to 5% canola oil or flaxseed oil in dual-purpose prime lambs had no negative effect on growth performance and wool quality. Moreover, the inclusion of 5% canola oil in pellets increased CG gain. Lamb breed significantly affected CG gain and wool quality. First-cross WxC lambs had the greatest CG gain while purebred Merinos had the best wool quality. Ewes had significantly greater FD than wethers, although gender did not influence other characteristics. Moderate to very strong correlations detected between wool quality traits were significant. In conclusion, canola and flaxseed oils can be effectively used in dual-purpose sheep systems during the 10-week feedlot period. The observed interaction effects of breed with oil supplementation permit flexibility in operational options of optimising profitability from meat in dual-purpose lamb production. It is proposed that supplementing 5% canola oil in CxM lamb diets or 5% flaxseed oil in WxC lamb diets could considerably improve their growth performance without detrimental impacts on wool quality, a win-win case of nutrient partitioning into the synthesis of muscle and wool without compromising either trait.
